# Predicting criminal and violent outcomes in psychiatry: a meta-analysis of diagnostic accuracy

**DOI:** 10.1038/s41398-022-02214-3

**Published:** 2022-11-09

**Authors:** Devon Watts, Taiane de Azevedo Cardoso, Diego Librenza-Garcia, Pedro Ballester, Ives Cavalcante Passos, Felix H. P. Kessler, Jim Reilly, Gary Chaimowitz, Flavio Kapczinski

**Affiliations:** 1 Department of Psychiatry and Behavioural Neurosciences, McMaster University, Hamilton, ON Canada; 2 Neuroscience Graduate Program, McMaster University, Hamilton, ON Canada; 3 Post-Graduation Program in Psychiatry and Behavioural Sciences, Federal University of Rio Grande do Sul (UFRGS), Porto Alegre, RS Brazil; 4 Laboratory of Molecular Psychiatry, Hospital de Clínicas de Porto Alegre (HCPA), Porto Alegre, RS Brazil; 5 Instituto Nacional de Ciência e Tecnologia Translacional em Medicina (INCT-TM), Porto Alegre, RS Brazil; 6 Center for Drug and Alcohol Research, HCPA, Porto Alegre, RS Brazil; 7 Department of Electrical and Computer Engineering, McMaster University, Hamilton, ON Canada; 8 Forensic Psychiatry Program, St. Joseph’s Healthcare Hamilton, Hamilton, ON Canada

**Keywords:** Schizophrenia, Predictive markers

## Abstract

Although reducing criminal outcomes in individuals with mental illness have long been a priority for governments worldwide, there is still a lack of objective and highly accurate tools that can predict these events at an individual level. Predictive machine learning models may provide a unique opportunity to identify those at the highest risk of criminal activity and facilitate personalized rehabilitation strategies. Therefore, this systematic review and meta-analysis aims to describe the diagnostic accuracy of studies using machine learning techniques to predict criminal and violent outcomes in psychiatry. We performed meta-analyses using the mada, meta, and dmetatools packages in R to predict criminal and violent outcomes in psychiatric patients (*n* = 2428) (Registration Number: CRD42019127169) by searching PubMed, Scopus, and Web of Science for articles published in any language up to April 2022. Twenty studies were included in the systematic review. Overall, studies used single-nucleotide polymorphisms, text analysis, psychometric scales, hospital records, and resting-state regional cerebral blood flow to build predictive models. Of the studies described in the systematic review, nine were included in the present meta-analysis. The area under the curve (AUC) for predicting violent and criminal outcomes in psychiatry was 0.816 (95% Confidence Interval (CI): 70.57–88.15), with a partial AUC of 0.773, and average sensitivity of 73.33% (95% CI: 64.09–79.63), and average specificity of 72.90% (95% CI: 63.98–79.66), respectively. Furthermore, the pooled accuracy across models was 71.45% (95% CI: 60.88–83.86), with a tau squared (τ^2^) of 0.0424 (95% CI: 0.0184–0.1553). Based on available evidence, we suggest that prospective models include evidence-based risk factors identified in prior actuarial models. Moreover, there is a need for a greater emphasis on identifying biological features and incorporating novel variables which have not been explored in prior literature. Furthermore, available models remain preliminary, and prospective validation with independent datasets, and across cultures, will be required prior to clinical implementation. Nonetheless, predictive machine learning models hold promise in providing clinicians and researchers with actionable tools to improve how we prevent, detect, or intervene in relevant crime and violent-related outcomes in psychiatry.

## Introduction

Available evidence suggests that one in eight men, and one in sixteen women will subsequently commit a serious criminal offense after release from a psychiatric facility [1]. This phenomenon is not isolated to specific geographical or generational effects, considering that in a systematic review comprising 33,588 individuals from 24 countries and 109 datasets, high rates of mental illness in prisoners were found in both high- and low-income countries over the timespan of four decades [2].

Additionally, results from a large Swedish registry study comprising 98,082 individuals with a history of hospitalization suggests that one in every twenty violent crimes is committed by someone with severe mental illness [3]. Given the high prevalence of criminal acts committed across cultures in individuals with severe mental illness, there has been a concerted effort to identify predictors of prospective criminal risk following discharge from psychiatric facilities.

In response to this, actuarial assessments became increasingly widespread, which use statistical algorithms to identify prospective patient risk, usually at the group level [4]. However, there is little evidence that actuarial risk estimates can accurately determine whether a specific patient will reoffend or commit subsequent acts of violence [5]. This is largely because most risk estimates have been developed statistically to assess group-based risk and perform poorly when making individualized predictions [5]. Altogether, this illustrates the limitations of current methods and the importance of a more precise, effective, and personalized approach to risk assessment in forensic settings. Given the ethical, psychiatric, and legal ramifications of inappropriately mischaracterizing the prospective risk of any given patient, and the resulting consequences to the individual, their families, and broader society, there is a growing interest in the use of artificial intelligence and predictive analytics to facilitate clinical decision making at an individual level [6]. This can potentially pave the way for tailor-made tools for the diagnosis, assessment, and treatment of patients [7, 8]. While predictive machine learning models have already shown promise in other fields of medicine [9, 10], there is a growing effort towards predicting criminal outcomes in psychiatric patients at an individual level. Incorporating such models into routine clinical care presents the potential to facilitate personalized and targeted rehabilitation strategies to decrease prospective criminal outcomes. To the best of our knowledge, there are no systematic reviews describing the diagnostic accuracy of machine learning models in predicting criminal and violent outcomes in psychiatry. Therefore, this systematic review and meta-analysis aim to assess the diagnostic accuracy of studies using machine learning techniques to predict criminal outcomes in psychiatry.

## Methods

This study has been registered on PROSPERO with the registration number PROSPERO CRD42019127169.

### Search strategy

We searched three electronic databases (PubMed, Scopus, and Web of Science) for articles published up until April 2022. To identify relevant studies, the following structure for the search terms was used: (Artificial Intelligence OR Supervised Machine Learning AND crime-related outcomes in psychiatry). The complete search filter is available in the supplementary material. We also screened references from included articles to search for potentially missed articles.

### Eligibility criteria

This systematic review was performed according to the PRISMA statement [11]. We selected original articles that used supervised machine learning models to predict crime-related outcomes in mental illness. We excluded review articles and studies using unsupervised learning, since methods such as clustering are not outcome oriented. Furthermore, studies that predicted crime or violent-related outcomes in individuals without psychiatric disorders were excluded, although further information regarding these studies can be found in Supplementary Table 2.

### Data collection and extraction

Potential articles were independently screened in a blinded standardized manner for title and abstract contents by two researchers (DW and DLG). Following this, the full texts of screened articles were obtained and evaluated according to the inclusion and exclusion criteria. A third author (PB) provided a final decision in cases of disagreement. Criminal outcomes were operationalized as rearrest, reconviction of crimes, or prediction of the type of crime committed. Violent outcomes involved recorded violent incidents during inpatient stay or following hospital discharge.

### Quality assessment

We created a machine learning quality assessment table based on experts’ opinion to evaluate the reproducibility and reliability of the included studies. Our assessment provides a quick way to evaluate published papers and can also serve as a checklist for future studies. Briefly, the instrument comprises nine methodological considerations, including representativeness of the sample, confounding variables, outcome assessment, algorithm selection, feature selection, class imbalance (where applicable), missing data, performance/accuracy, and testing/validation. The instrument can be found in Supplementary Table S1, and further details can also be found in the Supplementary Material.

### Statistical analysis

A bivariate meta-analysis was performed for crime-related and violent outcomes using the mada [6] meta [12], and dmetatools packages in R [6]. Since we anticipated considerable between-study heterogeneity, a random effects model was used to pool effect size. Additionally, an adjusted profile restricted maximum likelihood estimator was used to calculate the heterogeneity variance tau square (τ^2^). This metric was selected since the heterogeneity statistic ***I***^**2**^ can be biased in meta-analyses with small sample sizes [13]. Using the retisma function in ‘mada’ [6], a linear mixed model with random effects was selected to produce summary estimates of sensitivity and specificity, as well as calculate AUC and partial AUC summary receiver operating characteristic (ROC) curves, as described elsewhere [14]. 95% confidence intervals for summary AUC were generated using 2000 iterations of parametric bootstrapping with the ‘dmetatools’ package in R. Additionally, using the metamean function in ‘meta’ [12], mean accuracy across models was pooled alongside standard error of model accuracy, as detailed in Supplementary Table S3. As we anticipated considerable between-study heterogeneity, a random effects model was selected to pool effect sizes. The restricted maximum likelihood estimator [15] was selected to calculate the heterogeneity variance τ2. Knapp-Hartung adjustments [16] were also used to calculate the confidence interval around the pooled effect. Additionally, we pooled the diagnostic odds ratio, and the positive negative and likelihood ratios within a random effects model with a DerSimonian-Laird estimator [17].

Four studies were excluded from the meta-analysis, as the authors did not report the sensitivity and specificity of their models. Criminal outcomes were operationalized as rearrest, reconviction of crimes, or prediction of the type of crime committed. Violent outcomes involved recorded violent incidents during inpatient stay or following hospital discharge.

## Results

**Fig. 1:**
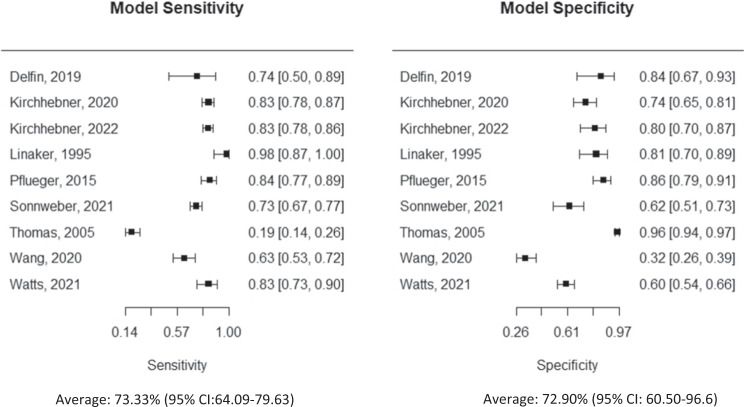
Paired Forest plot of model accuracy for criminal and violent outcomes in psychiatry. A linear mixed model with random effects was selected to produce summary estimates of sensitivity and specificity using the retisma function in mada. The average sensitivity across studies was 73.33% (95% I: 64.09–79.63) and average specificity was 72.90% (95% CI: 60.50–96.6). As such, the balanced accuracy across models (sensitivity + specificity/2) is 73.11%.

We found 12,420 potential titles/abstracts and included 20 studies which met inclusion criteria. A list of the included studies and their most relevant characteristics and findings are described in Table 1, while Table 2 details the diagnostic accuracies, odds ratios, and likelihood ratios of studies contained within the meta-analysis. Additionally, a schematic of the meta-analytic diagnostic accuracy of predicting criminal recidivism and physical violence are detailed in Fig. 1. Furthermore, a machine learning quality assessment, additional figures related to model performance, and a table comprising twenty-one studies assessing criminal outcomes in non-psychiatric individuals can be found in the supplementary material. Additional information about machine learning algorithms [18] including methodological considerations, common problems, and limitations, can be found elsewhere [19].

**Table 1:** Predicting criminal and violent outcomes in psychiatry.

First author, year	Data utilized	Outcome	Sample size and diagnosis^a^	Validation	Machine learning model	Accuracy	Other measures
CRIMINAL OUTCOMES							
Cohen et al. [20]	Clinical and administrative data	Subsequent arrest	127 male patients found not guilty by reason of insanity	N/A	Stepwise discriminant analysis	76%	N/A
Delfin et al. [21]	resting-state regional cerebral blood flow (rCBF) and clinical risk factors	Criminal recidivism	44 forensic psychiatry patients	Out-of-bag (OOB) error	RF	Accuracy: 82%	AUC: 0.81 Sensitivity: 75% Specificity: 86% PPV = 0.73 NPV = 0.86 Note: the dataset was not split into training and testing sets, and OOB error was used as a resampling procedure
Falconer et al. [22]	Age, past arrests, mental health diagnosis, enrollment to the JDP as well as utilization of outpatient group services, medical services, and case management	Rearrest	2100 adult offenders with records in US mental health services and the criminal justice system	Training (80%) and testing (20%) sets	Elastic Net regularized logistic regression	N/A	AUC (test set) 0.67 0.60 (simplified model)
Grann and Lngström [23]	10 risk factors of the Historical subscale of the HCR-20	Reconviction for a violent crime	404 violent offenders with a mental disorder followed up to eight years	Holdout validation with training/testing (2:1) (ANN)	BLR MLR ANN	N/A	AUCs BLR: 0.66–0.77 MLR: 0.63–0.73 ANN: 0.51–0.73
Kirchebner et al. [24]	Sociodemographic, clinical, behavioral, and symptom variables	Criminal recidivism	344 offenders with schizophrenia	Training (70%) and testing (30%) sets	Boosted Trees Naive Bayes	67.6–79.4% *Best performance using Naive Bayes*	*Best model* Naive Bayes with imputation AUC: 0.83 Sensitivity: 83% Specificity: 74% PPV: 84% NPV: 73%
Pflueger et al. [25]	Demographic variables and clinical scales (Basel Catalog for Risk Assessment, Historical Clinical Risk Assessment, and the Psychopathy Checklist- screening version)	Risk of general criminal recidivism of offenders with mental illness	259 individuals subjected by court orders to forensic psychiatric evaluation for mental and behavioral disorders using the ICD-10	4-fold cross-validation	RF	Best model had an overall 85% accuracy and accounted for 91% of all observed re-offenses.	Best model had a sensitivity of 84% and specificity of 86%.
Sonnweber et al. [26]	Clinical, developmental and social factors	Discriminating between violent and nonviolent offending	370 forensic offenders with schizophrenia	Training (70%) and testing (30%) sets	LR RF GBM KNN SVM Naive Bayes	Best model had a balanced accuracy of 67.82%	Sensitivity: 72.73% Specificity: 62.92% PPV: 65.98 NPV: 70.00 AUC: 0.764
Watts et al. [27]	Sociodemographic, clinical, behavioral, and symptom variables	Type of criminal offence (violent, sexual, nonviolent)	1240 transdiagnostic patients	Training (70%) and testing (30%) sets	RF Elastic Net SVM	Violent vs Sexual Offences: 65.27–80.31% Non-violent vs Sexual Offences: 49.56–77.62% Sexual Offences vs Violent and Non-violent: 59.82–71.58%	Best models: Violent vs Sexual Offences: Sensitivity: 76.74% Specificity: 83.87% PPV: 97.06 NPV: 34.21 Non-violent vs Sexual Offences: Sensitivity: 74.60% Specificity: 80.65% PPV: 80.65% NPV: 60.98% Sexual vs Non-violent and Violent Offences: Sensitivity: 83.15% Specificity: 60.00% PPV: 95.08 NPV: 27.69
VIOLENT OUTCOMES							
Kirchebner et al. [40]	Clinical variables pertaining to childhood, adolescence, adulthood and psychiatric stressors	Violent offending in schizophrenia	370 offenders with schizophrenia	5-fold cross-validation; no external validation used.	Boosted Classification Trees	76.4%	Sensitivity: 80.49 Specificity: 71.19 PPV: 66 NPV: 84 AUC: 0.83
Le et al. [31]	Text analysis from electronic mental health records	Forensic risk assessment ratings as a proxy of violence to others	Four NLP dictionary word lists - 6865 mental health symptom words from Unified Medical Language System, 455 DSM-IV diagnoses from UMLS repository, 6790 English positive and negative sentiment words, and 1837 high-frequency words from the Corpus Contemporary American English (COCA). *Exact number of patients not reported*	10-fold stratified cross-validation; no external validation used.	Bagging J48 JRip LMT LR Linear Regression SVM	SVM and LMT were the most accurate algorithms (accuracy of 69–77%) with all three dictionaries.	N/A
Linaker and Iversen [32]	55 items describing symptoms or behaviors reported or believed to be positively or negatively related to violent behaviors, obtained through screening of the medical records in the 24 h prior to the outcome	Physical violence towards others, assessed by the screening of medical records	94 patients admitted to a maximum-security psychiatric unit	Holdout validation, with training (46.1%) and testing (53.9%) sets	LR	92.1%	Specificity 100% Sensitivity 81.3%
Menger et al. [28]	25.942 doctor and nurse text notes at the start of admission (predictors) and violence incident reports (outcome)	Violent incidents in an inpatient unit occurring within the first 30 days of admission	2521 psychiatric admissions from 6 inpatients units	5-fold cross-validation; no external validation	RNN CNN NN NB SVM DT	N/A	AUCs ranged from 0.654 (word embeddings with RNN) to 0.788 (documents embedding with RNN)
Menger et al. [29]	Electronic health records	Inpatient violent risk	2209 psychiatric patients	Training (53.5%) and testing (46.5%) samples	SVM (radial kernel)	Testing / Validation (Sensitivity/Specificity) Site 1: 92.5% / 24.8% Site 2: 92.9% / 13.4%	AUC Site 1: 0.722 (0.690–0.753 95% CI) Site 2: 0.643 (0.610–0.675 95% CI)
Monahan et al. [33]	Clinical data obtained from interview, records, and questionnaires	Violent incidents after 20 weeks of hospital discharge	939 psychiatric inpatients	Bootstrapping	ICT	N/A	72.6% of the sample classified as low or high risk based on the prevalence of incident events based on a cut-off stipulated by the authors
Steadman et al. [34]	Clinical and demographic risk factors collected through the MacArthur Violence Risk Assessment Study	Predictors of violence risk	939 psychiatric patients assessed during the first 20 weeks following hospital discharge	Bootstrapping (1000 random samples with replacement drawn from original sample of 939).	LR CTA ICT	N/A	LR: 0.81 AUC CTA: 0.79 AUC ICT: 0.82 AUC Did not report sensitivity, specificity, PPV or NPV.
Suchting et al. [30]	Demographic variables, psychometric variables, and saliva samples for genetic testing of FKBP5 SNPs (FKBP5_13 (rs1360780); FKBP5_92 (rs9296158); and FKBP5_94 (rs9470080).	Predictors of State Aggression in individuals with previous trauma	48 participants selected irrespective of DSM diagnostic or psychometrically established clinical cut-offs for trauma exposure.	10-fold cross-validation; no external validation used.	Component-wise gradient boosting; backward elimination used for feature selection.	N/A	8-factor model *R*^2^ = 0.66 Did not report AUC, accuracy, sensitivity, specificity, PPV or NPV.
Suchting et al. [35]	Extracting variables using retrospective electronic health records	Predictors of aggression in inpatients	29,841 patient records from the Harris County Psychiatric Center	10-fold cross-validation; no external validation used	Four different algorithms: GLM RF GBM DNN	N/A	GLM: 0.7801 AUC RF: 0.7420 AUC GBM: 0.7765 AUC DNN: 0.7137 AUC
Thomas et al. [36]	Data from a large randomized controlled trial in 4 inner-city mental health services in the United Kingdom (clinical/demographic variables)	Predictors of violence among patients with psychosis	780 patients with psychosis, 158 of which were violent during the 2-year follow-up period	10-fold cross-validation; no external validation used	Full logistic regression (14 variables) Forward stepwise logistic regression (6 variables) Full CART (123 nodes) Pruned CART (22 nodes) Pruned CART (22 nodes: violent cases given, 5 x weight)	57.5%	*Best Performance* Full logistic regression Sensitivity - 19% Specificity - 96% PPV - 49% NPV - 79% Percent correctly classified - 77%
Tzeng et al. [37]	Patient insight ratings, medication compliance, and demographic characteristics Schedule for Assessment of Insight in Psychosis (SIP) Violence and Suicide Assessment Scale (VASA)	Presence or absence of violent behavior towards people or things (1 year later)	63 outpatients with schizophrenia, according to the DSM-IV, who were in remission or had minimal psychosis symptoms	3-fold cross-validation; no external validation used	SVM	76.2%	An LR model was used as a point of comparison, however, no resampling measures were used (model developed using the entire sample)
Wang et al. [38]	Identified 28 variables previously identified with violence or schizophrenia (Structured interviews, self-report questionnaires, medical history, and demographic information)	Violent vs Non-violent (Ranging from absence of physical violence to assault causing bodily harm according to the Modified Overt Aggression Scale)	275 patients with schizophrenia spectrum disorder, according to the DSM-IV	5-fold cross-validation; no external validation used	LR LASSO Elastic Net RF GBRT SVM *radial kernel*	57–62% *Best performance using RF*	*Best performance* *Random Forest* AUC: 0.63 (±0.004) Sensitivity: 63% (±0.005) Specificity: 32% (±0.008) PPV: 62% (±0.008) NPV: 54% (±0.003)

A summary of input data, sample characteristics, validation methods, and machine learning models across studies.

*ANN* Artificial neural networks, *AUC* Area under the curve, *CART* Classification and regression trees, *CNN* Convolutional neural networks, *CTA* Classification Tree Analysis, *DNN* Deep neural networks, *DSM IV-R* Diagnostics and Statistical Manual, Version IV, Revised, *DT* Decision tree, *EN* elastic net, *GBRT* Gradient Boosted Regression Trees, *HCR-20* Historical, clinical, risk management-20, *ICT* Iterative classification tree, *LASSO* Least Absolute Shrinkage and Selection Operator, *LR* Logistic regression, *NB* Naive Bayes, *NN* Neural network, *NPV* Negative Predictive Value, *PPV* Positive Predictive Value.

^a^The sample size shown in the table includes only the number of subjects used for the machine learning model development, with subjects used for other purposes, such as statistical analysis, not being included in this number.

**Table 2:** Performance Metrics: Accuracies, AUC, diagnostic odds ratio, and likelihood ratios.

(a)						
Authors	Sensitivity	2.5%	97.5%	Specificity	2.5%	97.5%
Delfin [21]	0.750	0.498	0.886	0.845	0.674	0.935
Kirchebner, [24]	0.830	0.777	0.873	0.739	0.651	0.811
Kirchebner [40]	0.826	0.780	0.865	0.801	0.700	0.875
Linaker [32]	0.985	0.870	0.998	0.811	0.696	0.890
Pflueger [25]	0.841	0.768	0.894	0.860	0.790	0.909
Sonnweber [26]	0.727	0.674	0.775	0.625	0.513	0.725
Thomas [36]	0.545	0.415	0.673	0.823	0.795	0.850
Wang [38]	0.630	0.534	0.716	0.321	0.256	0.394
Watts [27]	0.873	0.785	0.961	0.605	0.459	0.751
AVERAGE	0.733	0.640	0.796	0.729	0.639	0.796
Test for equality of sensitivities: **X-squared** = 281.09, *p*-value = <0.000001						
Test for equality of specificities: **X-squared** = 382.63, *p*-value = <0.000001						
Correlation of sensitivities and false positive rates: Rho = 0.150 (-0.571–0.740)						
**Total DOR**: 9.57 (95% CI: 4.03–22.72), τ2 = 9.57 (95% CI: 0.00–6.93)						
**Log DOR**: 2.466 (95% CI: 1.534–3.397)						
**posLR**: 3.083 (95% CI: 1.954–4.866), τ2 = 0.437 (0.000–0.947)						
**negLR**: 0.342 (95% CI: 0.201–0.583), τ2 = 0.566 (0.000–0.3476)						
**AUC**: 0.816 (95% CI: 0.745–0.875); **pAUC**: 0.733						

(b)			
Authors	Mean	95% CI	%W (random)
Delfin, [21]	80.50	68.92–94.02	10.1
Kirchebner [24]	79.40	76.04–82.90	11.5
Kirchebner [40]	75.84	71.65–80.27	11.4
Linaker [32]	90.65	83.78–98.07	11.2
Pflueger [25]	85.00	80.62–89.60	11.4
Sonnweber, [26]	67.82	61.31–75.01	10.9
Thomas [36]	57.50	51.20–64.57	10.7
Wang [38]	47.00	46.53–47.46	11.6
Watts [27]	71.58	67.04–76.42	11.3
AVERAGE	71.45	60.88–83.85	100%
Number of Observations: 2798, τ^2^ = 0.042 (95% CI: 0.018–0.153)			

(a) Using the retisma function in mada, a linear mixed model with random effects was selected to produce summary estimates of sensitivity and specificity, as well as calculate AUC and partial AUC summary receiver operating characteristic (ROC) curves. Spearman’s rho was used to assess correlation between sensitivities and false positive rates of included studies. The total diagnostic odds ratio (DOR), and positive and negative likelihood ratios (posLR, negLR) were calculated in a random effects model with a DerSimonian-Laird estimator using the maduani function in mada. The 95% confidence interval (CI) for AUC was calculated using bootstrapping with 2000 iterations with the dmetatools package in R. The average AUC across models was 0.816 (95% CI: 0.745–0.875), with a partial AUC of 0.733, and log DOR of 2.466 (95% CI: 1.534–3.397).

(b) Using the metamean function in meta, the pooled accuracy of criminal and violent models was performed across 2428 patients (two studies used the same sample *n* = 370) within a random effects model using a restricted maximum likelihood estimator to calculate the variance τ^2^. Knapp-Hartung adjustments were used to calculate the confidence interval around the pooled effect. The average accuracy across models was 71.45% (95% CI: 60.88–83.86), with a heterogeneity variance τ^2^ of 0.0424.

Of the studies included in the systematic review, six assessed predictors of criminal recidivism [20–25], two assessed predictors of the type of criminal offence [26, 27], three assessed predictors of physical violence during inpatient stay [28–30], and six assessed predictors of violent offending and aggression following discharge [24, 31–38]. All studies, apart from two [21, 30], used clinical input features, including socio-demographic information, questionnaires, and psychometric measures to derive predictions.

### Studies assessing criminal outcomes

Eight studies used machine learning models to predict criminal outcomes in patients with psychiatric disorders [20–27]. Delfin and colleagues conducted the first 10-year follow-up of a cohort of forensic psychiatry patients, including 44 individuals, who underwent a single-photon emission CT scan. These data, alongside eight evidence-based clinical risk factors, were used in a random forest model to predict criminal recidivism, resulting in an accuracy of 82% and an AUC of 0.81. Of note, when only clinical risk factors were used alone, model performance degraded, with an accuracy of 64% and AUC of 0.69, emphasizing the importance of combining clinical and biological features to predict criminal recidivism. The top features reflecting neuronal activity included the right and left parietal lobe, left temporal lobe, and right cerebellum [21].

Kirchebner and colleagues used 653 clinical features to predict recidivism in 344 individuals with schizophrenia. Patients who had a criminal record prior to their current offence were considered as recidivists. Following imputation, the best performance was observed using Boosted Trees, with an accuracy of 67.6%. Without imputation, a Naive Bayes classifier achieved an accuracy of 79.4%. Important variables included amisulpride prescription prior to offence, recent stressors, recent legal complaints, and number of prior offences [24].

Sonnweber et al. developed a model to differentiate between violent and non-violent offenders in patients with schizophrenia. The best performance was observed using a gradient boosting machine, resulting in a balanced accuracy (operationalized as the average of sensitivity and specificity, as defined elsewhere [39]) of 67%. The most important variables included time spent in hospitalization, age at diagnosis, daily olanzapine at discharge, PANSS score at discharge, and social isolation in adulthood [26].

Furthermore, Watts and colleagues developed a machine learning model to predict the type of criminal offence committed in a large transdiagnostic sample of 1240 psychiatric patients. Using multiclass classification, they showed that sexual crimes could be discriminated from violent and nonviolent crimes at an individual level with an accuracy of 71.22%. Moreover, following recursive feature elimination, a reduced model with 36 variables resulted in an accuracy of 71.58%. The most important features for the model included previous absolute discharge, previous sexual convictions, cluster A personality disorder, and female gender [27]. Other studies predicted rearrest after release from jail [20, 22], reconviction for a violent crime [23], and risk of general criminal recidivism [25]. A summary of these findings can be found in Table 1 and Supplementary Table S2.

### Studies assessing violent outcomes

Twelve studies used machine learning techniques to predict violent outcomes in patients with psychiatric disorders [28–38, 40]. Linaker and colleagues predicted violent incidents in psychiatric patients using behavioral symptoms from health records from 24 h prior. Overall, 48 acts of violence were recorded from 32 patients, and following feature selection using correlation coefficients, six variables were used as predictors in a logistic regression model. The authors reported a sensitivity of 81.3% and specificity of 100%, however it was unclear how class imbalance was addressed, since only 34.7% of patients committed an act of violence during the study [32].

Kirchebner and colleagues used a series of known stressors to predict violent offending in 370 patients with schizophrenia. The overarching goal was to determine whether accumulated stressors precipitated violent outcomes in patients. Using boosted classification trees, they reported an accuracy of 76.4%. However, no external validation or testing set was used, instead, performance was assessed using 5-fold CV [40].

Furthermore, Menger et al. used text analysis from doctor and nurse notes to predict violent incidents in psychiatric inpatients. Four feature extraction methods were used, comprising binary bag of words, term frequency-inverse document frequency (tf-idf) bag of words, document embeddings, and word embeddings, as described elsewhere. An AUC of 0.788 was observed using document embeddings with recurrent neural networks. The worst performances occurred with the Naive Bayes algorithm, which is the most classical and widely used algorithm for text classification [28].

Monahan and colleagues classified patients according to high and low risk of violence following discharge from psychiatric facilities. Decision trees were used in a binary classification task, and features were selected using a stepwise model, where the threshold of statistical significance between the feature and outcome were set at *P* < 0.05. The model correctly identified 72.6% of the sample as either low or high risk. Important variables included seriousness of prior arrests, motor impulsiveness, paternal drug use, and recurrent violent fantasies. It is important to mention that 27.4% of the total sample remained unclassified, meaning it could find no combination of risk factors to classify patients into high or low-risk groups [33].

Additionally, Suchting and colleagues used saliva FK506 binding protein 5 (FKBP5) polymorphisms alongside demographic and psychometric variables to predict state aggression, which resulted in an *R*^2^ of 0.66 [30]. Other studies identified predictors of violent risk following discharge [37, 38] and aggression in patients [29, 31, 34–36], which are further described in Table 1.

### Meta-analysis of diagnostic accuracy

**Fig. 2:**
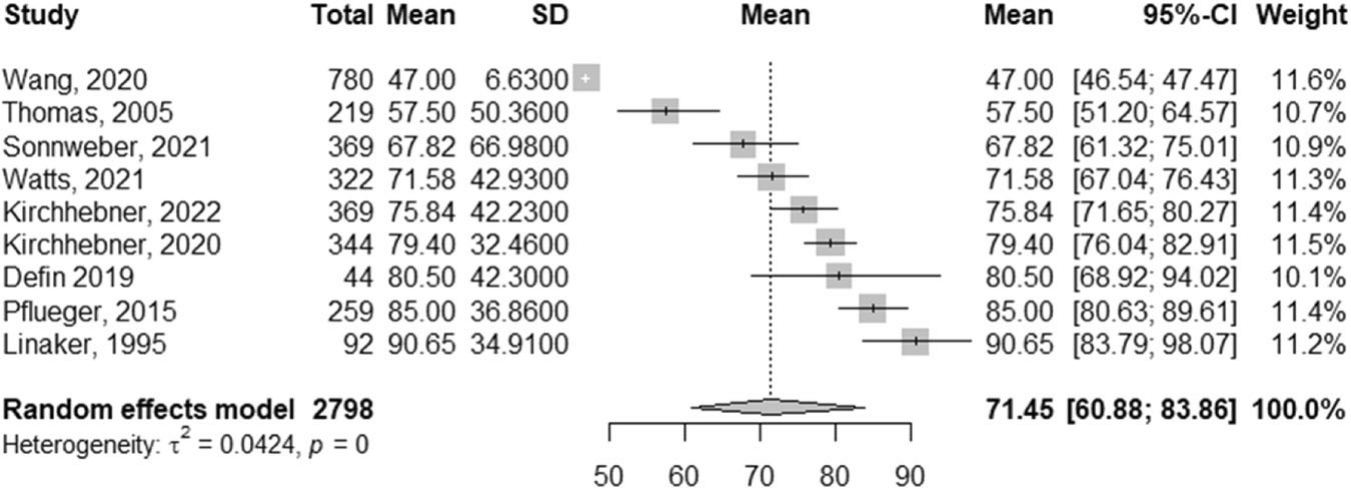
Pooled effects of model accuracy. Pooled accuracy of criminal and violent models in psychiatry across 2428 patients (two studies used the same sample *n* = 370) within a random effects model using a restricted maximum likelihood estimator to calculate the heterogeneity variance τ2. Reported mean accuracy across models was used, in conjunction with standard deviation, calculated by multiplying the standard error by the square root of the sample size (SD = SE×√*n*). Knapp-Hartung adjustments were used to calculate the confidence interval around the pooled effect. The average accuracy across models was 71.45% (95% CI: 60.88–83.86), with a heterogeneity variance τ^2^ of 0.0424.

A forest plot detailing model performance can be observed in Figs. 1 and 2, while Table 2 details the diagnostic accuracies, odds ratios, and likelihood ratios across studies. Additional details related to the standard error of model accuracy, 95% CI, and the true/false positives and negatives, can be found in Supplementary Table S3. Nine studies were pooled, comprising 2,428 patients (the same dataset of 370 patients was used across two studies [26, 40]).

Additionally, nine studies which did not report the sensitivity and specificity of models [20, 22, 23, 28, 29, 31, 33–35], and one regression-based model [30] were excluded from the meta-analysis. Overall, the pooled accuracy across models was 71.45% (95% CI: 60.88–83.85), with a sensitivity ranging from 54.4%-87.3% (average: 73.33%, 95% CI: 64.09–79.63) and specificity ranging from 60.5–96.6% (average: 72.90%, 95% CI: 63.98–79.66). The heterogeneity statistic τ^2^ for pooled model accuracy was 0.0424 (95% CI: 0.0184–0.1553). A plot of the false positive rate against sensitivity for all studies can be found in Supplementary Fig. S1.

The diagnostic odds ratio (DOR) across studies was 9.75 (95% CI: 4.035–22.72; τ^2^ = 1.505) as detailed in Table 2. Similarly, the positive likelihood ratio (posLR) was 3.083 (95% CI: 1.954–4.866, with a τ^2^ of 0.437 (95% CI: 0.000–0.897), and the negative likelihood ratio (negLR) was 0.342 (95% CI: 0.201–0.583), with a τ^2^ of 0.566 (95% CI: 0.000–3.476), respectively. Additionally, the log DOR across studies was 2.466 (95% CI: 1.534–3.397). The average prevalence of the positive class (presence of criminal and violent outcomes) was 43.435% of the sample across studies. Furthermore, the AUC across studies was 0.816 (95% CI: 0.745–0.875) in predicting criminal and violent outcomes, with a partial AUC of 0.773. Spearman’s rho indicated a weak association (rho = 0.150, 95% CI: −0.571–0.740) with a large confidence interval between the sensitivities and false positive rates of included studies.

## Discussion

To the best of our knowledge, this is the first systematic review comprising studies using supervised machine-learning techniques to predict criminal or violent outcomes in individuals with psychiatric disorders. Throughout our review, we have identified recurrent features and algorithms used, as well as current methodological challenges. In this section, we detail key aspects of these models, showcasing their limitations as well as our perspectives on best practices for developing machine learning models with clinical utility. Further details regarding common methodological issues in machine learning models can be observed in the supplementary material.

### Model interpretability, model performance, and confidence intervals

More recent machine learning algorithms that use regularization parameters to account for common issues such as multicollinearity, tended to show higher performance accuracy in predicting outcomes. However, model complexity carries the trade-off of greater difficulty in model interpretability and explainability [41].

Recently, new local explanation methods have been developed, including SHapley Additive exPlanations (SHAP), to explain variable contributions at the individual level [42]. Adaptations of this, such as TreeExplainer, leverage the internal structure of tree-based models to efficiently compute local explanations using Shapley values [43]. Moreover, SHAP dependence plots can be used to showcase the effect that a single feature has on predictions made by the model [43]. In two studies included in the current review, feature importance metrics were not reported [28, 35]. It is argued that future studies may benefit from an increased focus on model interpretability, which may aid in the generalizability and replicability of such work.

Furthermore, it is important to highlight that model performance can be over-optimistic when assessed using internal cross-validation alone, in the absence of separate training and testing sets. Of the twenty studies contained in the present review, only seven (35%) incorporated training and testing sets in model development. In the majority of studies [25, 28–31, 33–36, 38] (76.9%) that evaluated model performance using internal cross-validation alone, sample sizes were also well over 100 patients. As mentioned elsewhere, several other fields use cross-validation to tune regularization parameters in model development, rather than taking performance estimates at face value [44]. Similarly, it is important to mention that uncertainty estimates should be considered when evaluating model performance and its potential clinical utility. Of nine studies comprising the meta-analysis, only four (44.4%) [21, 26, 27, 37] reported accuracy estimates using a method such as 95% confidence intervals.

### Model performance and clinical predictors

Overall, eighteen models assessed clinical predictors of criminal and violent outcomes [20, 22–29, 31–38, 40]. In criminal prediction models, accuracy was generally high, ranging from 67.83–82%.

With respect to criminal behavior, common predictors across models included age at first crime, substance use disorder, cluster B personality disorder, prior criminality, a high number of stressors, and childhood trauma. Future work may benefit from comprising a standardized evidence-based risk battery for use in prospective models.

Furthermore, models predicting violent behavior were more variable, ranging from 58.25–92.1%, with five of twenty studies (25%) [22, 23, 28, 35] comprising the systematic review only reporting AUC. As such, several were excluded from the meta-analysis. Nonetheless, important clinical features included confusion, irritability, threats, recently attacking objects, child abuse, physical neglect, and callous affect. Important search terms included aggressive, offered, angry, door, walk, arrest, offer emergency medication, and walked.

With respect to the meta-analysis comprising nine studies (*n* = 2428 patients), the pooled accuracy was 71.45% (95% CI: 60.88–83.86) in predicting criminal and violent outcomes. Moreover, as detailed in Table 2, the DOR was 9.757 (95% CI: 4.035–22.72; τ^2^ = 1.505) and log DOR was 2.466 (95% I: 1.534–3.397). As discussed elsewhere, the DOR is a measure of the effectiveness of a diagnostic test that is independent of prevalence [45]. A DOR of 9.757 represents a high ratio of the odds of the test being positive if the individual will commit prospective criminal and violent outcomes relative to the odds of the test being positive if the individual will not prospectively commit criminal and violent outcomes. However, a large upper and lower bound of the 95% CI was observed, and the log DOR suggests a more conservative test effectiveness. Similarly, the posLR was 3.083 (95% CI: 1.954–4.866), suggesting a small increase in the likelihood of committing violent and criminal outcomes in patients with a positive test. In addition, the negative likelihood ratio was 0.342 (95% CI: 0.201–0.583), suggesting a 20–25% decrease in the odds of committing violent and criminal outcomes in patients with a negative test result.

### Model performance and biological predictors

Furthermore, two models [21, 30] assessed biological predictors pertaining to saliva SNPs and resting-state regional cerebral blood flow. Although they contained small sample sizes and lacked external validation, both showed promising performance, corresponding to an R2 of 0.66, and accuracy of 82%, respectively. Important features included KBP5_14 (rs1460780), FKBP5_92 (rs9296158); and FKBP5_94 (rs9470080), right and left parietal lobe rCBF, left temporal lobe rCBF, and right cerebellum. Subsequent studies may benefit from replicating these findings and incorporating additional biological and physiological variables.

### Limitations

Currently, the field of predicting crime and violent-related outcomes using machine learning techniques remain in its infancy. As such, there is a lack of studies validating model performance using independent cohorts. Furthermore, it is important to note that model accuracy should be considered alongside several other factors, such as the input features used, the preprocessing pipeline, feature selection method, model optimization strategy, and the validation procedure. Furthermore, data-driven approaches to feature selection can be useful in many cases, since it does not require knowledge derived from pre-existing literature to manually select important variables [46–48]. Of note, the absence of a formalized feature selection strategy was observed across a subset of studies.

There are several available feature selection methods, with varying degrees of appropriateness depending on the application, as described elsewhere [47]. Furthermore, feature selection can be useful to improve the generalizability of models when applied to independent datasets [49]. Considering that predictive models applied to forensic healthcare can have significant legal repercussions - such as incorrectly identifying individuals as not criminally responsible when in fact they are, or the inability to detect malingering - it is paramount that we use the most optimal methods available for these purposes.

Additionally, only two studies developed separate models to assess potential differences in performance between men and women using the same variables, as described in the supplementary material. Rosselini et al. reported an AUC of 0.74 for men and an AUC of 0.82 for women in predicting violent crime [50]. Additionally, the same authors also investigated predictors of major violent crime and reported an AUC of 0.81 for both models in men, and an AUC of 0.80–0.82 for both models in women. Based on these studies, it is still unclear whether biological sex or gender play a key role in deciding which features should be included within a predictive machine-learning model.

### Future directions

Moving forward, a further refinement of predictive models in forensic risk prediction is required. Potentially, this may be facilitated by using a wider framework when selecting the input data in our models. Considering that our model performance is directly dependent on the available input data, an exploratory data-driven approach may be warranted in predictive models.

Most machine learning studies in forensic psychiatry thus far focus purely on clinical and administrative data, given the widespread availability of such data. However, other modalities, such as neuroimaging (MRI, fMRI, DTI), electrophysiology (EEG, MEG, ERG) various sensors (actigraphy, heart rate variability), and genomic features (whole genome sequencing, whole exome sequencing, and RNA sequencing) may prove to facilitate model performance, when used in conjunction with clinical data. Moreover, longitudinal studies with larger multicentric samples and adequate external validation are needed to translate proof-of-concept predictive models into applications to be used in clinical and legal settings. We hypothesize that such models may facilitate a more personalized approach to patient evaluation and risk management, provide greater precision in deriving a tailored treatment plan, and aid clinicians and the legal system in the decision-making process as it pertains to mentally disordered offenders. Ultimately, they may become critical tools to assist in prison sentencing, to determine fitness to stand trial, and to optimize the progress of individuals in the forensic system towards rehabilitation.

## Supplementary information

Supplementary Table S1

Supplementary Table S2

Supplementary Table S3

Supplementary Figure S1

Supplementary Material

## Data Availability

R scripts used to generate Figs. 1, 2, and Supplementary Fig. S1 can be found in the supplementary material.
