# Supplementary material for: Predicting criminal and violent outcomes in psychiatry: a meta-analysis of diagnostic accuracy
Source: Transl Psychiatry. 2022 Nov 9;12:470. doi: 10.1038/s41398-022-02214-3 (PMC9643469; doi:10.1038/s41398-022-02214-3)
Supplement: Supplementary file 1 — Supplementary Table S1 [file 41398_2022_2214_MOESM1_ESM.docx]

Quality Scores of All Studies

| **CRIMINAL OUTCOMES** | | | | | | | | | | |
| --- | --- | --- | --- | --- | --- | --- | --- | --- | --- | --- |
| Authors | Representative | Confounding | Outcome | ML | Feature Selection | Class imbalance | Missing data | Performance | Testing/  Validation | Overall Score |
| Cohen, 1988 | Yes | Yes | 1) | No | Yes | No | Yes | No | Yes | 6/9 |
| Delfin, 2019 | No | Yes | 1) | Yes | Yes | Yes | Yes | Yes | No | 7/9 |
| Falconer, 2014 | Yes | No | 1) | Yes | Yes | No | No | No | Yes | 5/9 |
| Grann, 2007 | Yes | No | 1) | Yes | No | No | No | No | Yes | 4/9 |
| Kirchebner, 2020 | No | Yes | 2) | Yes | No* | No | Yes | Yes | Yes | 6/9 |
| McDermott, 2006 | No | Yes | 2) | No | Yes | No | No | Yes | No | 3/9 |
| Pflueger, 2015 | Yes | No | 2) | Yes | Yes | No | No | Yes | No | 4/9 |
| Sonnweber, 2021 | No | Yes | 2) | Yes | Yes | Yes | Yes | Yes | Yes | 7/9 |
| Watts, 2021 | Yes | Yes | 2) | Yes | Yes | Yes | Yes | Yes | Yes | 8/9 |
| **VIOLENT OUTCOMES** | | | | | | | | | | |
| Authors | Representative | Confounding | Outcome | ML | Feature Selection | Class imbalance | Missing data | Performance | Testing/  Validation | Overall Score |
| Kirchebner, 2022 | No | Yes | 2) | Yes | No | Yes | Yes | Yes | No | 5/9 |
| Le, 2018 | Yes | No | 2) | Yes | No | Yes | Yes | No | Yes | 6/9 |
| Linaker, 1996 | No | No | 2) | No | Yes | No | No | Yes | No | 4/9 |
| Menger, 2018 | Yes | Yes | 2) | Yes | Yes | No | Yes | No | No | 6/9 |
| Menger, 2019 | Yes | Yes | 2) | Yes | Yes | No | Yes | No | Yes | 7/9 |
| Monahan, 2000 | Yes | Yes | 2) | No | Yes | Yes | Yes | No | No | 6/9 |
| Steadman, 2000 | Yes | Yes | 2) | No | No | Yes | Yes | No | No | 5/9 |
| Suchting, 2018a | No | Yes | 3) | Yes | Yes | Yes | Yes | Yes | No | 6/9 |
| Suchting, 2018b | Yes | Yes | 2) | Yes | No | Yes | Yes | No | Yes | 7/9 |
| Thomas, 2005 | Yes | Yes | 2) | Yes | Yes | No | No | Yes | No | 6/9 |
| Tzeng, 2004 | No | Yes | 2) | Yes | No | No | Yes | No | No | 3/9 |
| Wang, 2020 | No | Yes | 2) | Yes | Yes | No | Yes | Yes | No | 5/9 |

Supplementary Table S1: Quality of all studies

We created a machine learning quality assessment table based on experts' opinion to evaluate the reproducibility and reliability of the included studies. Our assessment provides a quick way to evaluate published papers and can also serve as a checklist for future studies. Briefly, the instrument comprises nine methodological considerations, including representativeness of the sample, confounding variables, outcome assessment, algorithm selection, feature selection, class imbalance (where applicable), missing data, performance/accuracy, and testing/validation. Further details can be found in the Supplementary Material.

* Kirchebner 2020: Feature selection was performed by ranking all variables, in order of importance, according to how often they were identified as top variables across backward selection, logistic regression, trees, SVMs and naive bayes. However, the exact way this was operationalized is unclear.
