# Supplementary material for: Predicting criminal and violent outcomes in psychiatry: a meta-analysis of diagnostic accuracy
Source: Transl Psychiatry. 2022 Nov 9;12:470. doi: 10.1038/s41398-022-02214-3 (PMC9643469; doi:10.1038/s41398-022-02214-3)
Supplement: Supplementary file 2 — Supplementary Table S2 [file 41398_2022_2214_MOESM2_ESM.docx]

Table 1 – Machine learning studies predicting criminal and violent outcomes in non-psychiatric individuals.

| **First author, year** | **Data utilized** | **Outcome** | **Sample size and diagnosis^1^** | **Validation** | **Machine learning model** | **Accuracy** | **Other measures** |
| --- | --- | --- | --- | --- | --- | --- | --- |
|  | | | | | | | |
| **VIOLENT BEHAVIOR** | | | | | | | |
| **Barzman, 2018** | Demographic variables, assessments of aggression, and static risk factors | Risk of school violence | 103 middle and high school students recruited through outpatient clinics, inpatient units, and emergency department | Nested 10-fold cross-validation | LR with L2 normalization | N/A | 91.02% (assessments only)  91.45% (assessments, clinical and sociodemographic data) |
| **Gardner, 1996** | Clinical record data | Violence was determined using incident reports from psychiatric, arrest, or criminal records and clinical interviews. | 784 subjects with a psychiatric diagnosis (schizophrenia, affective disorders, substance use disorders, personality disorders, and others) | Not cross-validated | CART | N/A | Sensitivity / Specificity  One-stage RT: 7.7% / 99.2%  One-stage NBR: 9.3% / 99.1%  Two-stage RT: 6.9% / 99.3%  Two-stage NBR: 6.9% / 99.5% |
| **Rosellini, 2018** | Pre-Post Deployment Study (PPDS) of the Army STARRS dataset | Risk of interpersonal violence | 7081 soldiers deployed to Afghanistan. | 10-fold cross-validation; no external validation. | Ensemble learning:  LR  (EN with varying mixing parameter penalties, two SR, APS, two DT methods, BART, SVM, GBM, and NN) | N/A | Predictive models developed for each outcome, including depression (AUC 0.88), generalized anxiety disorder (AUC 0.85), suicidality (AUC 0.86) and head injury (AUC 0.74).  Super learner AUC was 0.79 for anger attacks, 0.80 for being bullied or hazed, and 0.75 for getting into a fight.  The sensitivity, specificity, and balanced accuracy of the models were not reported. |
| **Thomas, 2005** | Data from a large randomized controlled trial in 4 inner-city mental health  services in the United Kingdom (clinical/demographic variables) | Predictors of violence among patients with psychosis | 780 patients with psychosis, 158 of which were violent during the 2-year follow-up period. | 10-fold cross-validation; no external validation used. | Full logistic regression (14 variables)  Forward stepwise logistic regression (6 variables)  Full CART (123 nodes)  Pruned CART (22 nodes)  Pruned CART (22 nodes; violent cases given, 5 x weight) | N/A | **Full logistic regression**  Sensitivity - 19%  Specificity - 96%  PPV - 49%  NPV - 79%  Percent correctly classified - 77%  **Forward Stepwise Logistic Regression**  Sensitivity - 12%  Specificity - 45%  PPV - 41%  NPV - 78%  Percent correctly classified - 76%  **Full CART**  Sensitivity - 21%  Specificity - 86%  PPV - 31%  NPV - 79%  Percent correctly classified - 71%  **Pruned CART**  Sensitivity - 14%  Specificity - 93%  PPV - 38%  NPV - 78%  Percent correctly classified - 71%  **Pruned CART** (22 nodes; violent cases given, 5 x weight)  Sensitivity - 19%  Specificity - 87%  PPV - 30%  NPV - 75%  Percent correctly classified - 71% |
| **CRIMINAL OUTCOMES** | | | | | | | |
|  | | | | | | | |
| **Ang, 2013** | Clinical questionnaires | Being charged or not charged for initial juvenile offending | 2,899 adolescents from four school geographic areas | Holdout validation | LR  DT  ANN  SVM | Testing / validation  LR: 94.50 / 95.20  DT: 96.64 / 97.46  ANN: 97.22 / 98.26  SVM: 94.16 / 94.95 | AUC  LR: 0.950  DT: 0.968  ANN: 0.973  SVM: 0.946 |
| **Brodzinski, 1994** | Clinical and demographic data | Differentiating criminal recidivists from non-recidivists | 778 juvenile probation cases | Training (90%) and testing (10%) samples | Discriminant analysis  ANN | 63% (discriminant)  99% (ANN) | N/A |
| **Caulkins, 1996** | Clinical and administrative data | Criminal recidivism | 3508 offenders during a two-year period following release from federal prison | Holdout validation with training (57.9%) and testing (41.9%) samples | LR  MNN | Eighteen variable model:  LR: 0.689  MNN: 0.699  Eleven variable model:  LR: 0.683  MNN: 0.689  Eight variable model:  LR: 0.673  MNN: 0.684 | N/A |
| **Cope, 2014** | sMRI coupled with clinical assessments and sociodemographic data | Distinguishing homicide offenders from non-offenders | 155 youth from a maximum-security facility | Two nested LOOCV | SVM with feature selection | 81.29% (feature selection)  78.06% (no feature selection) | With feature selection:  Specificity: 75.00%  Sensitivity: 82.22%  No feature selection:  Specificity: 70.00%  Sensitivity: 79.26% |
| **Liu, 2011** | HCR-20 questionnaire | Reconviction by violent offenses | 882 male prisoners in England and Wales prospectively followed by a mean follow-up time of 3.31 years (1.34-4.24) | Holdout validation, with training (50%, testing (25%) and validation (25%) sets | LR  CART  ANN | N/A | Train  LR: 0.72-0.75  CART: 0.67-0.71  MLPNN: 0.71-0.78  Test  LR: 0.63-0.68  CART: 0.60-0.66  MLPNN: 0.65-0.70  Validation  LR: 0.64-0.66  CART: 0.58-0.66  MLPNN: 0.64-0.70 |
| **Palocsay, 2000** | Nine clinical/demographic variables | Criminal recidivism among individuals released from prison | 10357 prisoners in two cohorts | Holdout validation with training (n=2620), testing (n=7382) and validation sets (n=355) | Linear regression  ANN | 1978 ANN: 69.23%  1978 Logistic regression: 66.73%  1980 ANN: 66.98%  1980 Logistic regression: 65.71%  1978/1980 ANN: 65.96%  1978/1980 Logistic regression: 64.29 | Recidivist correct (%)  1978 ANN: 41.26  1978 Logistic regression: 30.41  1980 ANN: 40.93  1980 Logistic regression: 30.53  1978/1980 ANN: 39.01  1978/1980 Logistic regression: 36.35  Non-recidivist correct (%)  1978 ANN: 85.89  1978 Logistic regression: 88.43  1980 ANN: 82.84  1978/1980 ANN: 82.15  1978/1980 Logistic regression: 81.07 |
| **Rosellini, 2016** | Clinical and administrative data from the Army STARRS dataset | first accusation of a major physical violent crime | 975 057 soldiers in the US Army in 2004–2009 | Training (975, 057) and independent testing sample (43,248); of 10-fold cross-validated  forward stepwise regression used for feature selection | Stepwise regression, random forests, penalized regressions | 0.80-0.82 AUC in the training dataset and 0.77 AUC in the validation dataset | Sensitivity, specificity, PPV and NPV were not reported.  In the training dataset, an AUC of 0.81 was observed among men and 0.80-0.82 among women |
| **Rosellini, 2017** | Clinical and administrative data Army STARRS dataset | Any crime with sufficient evidence to warrant an investigation | 25,966 men and 2728 women who committed a first founded minor violent crime | 10-fold cross-validation; external testing sample used | Stepwise and Penalized regression  RF | AUC was 0.79 (for men and women) in the 2004-2009 training sample and 0.74-0.82 (men-women) in the 2011-2013 test sample. | N/A |
| **Silver, 2000** | Official clinical and administrative information of offenders convicted of an indictable offense | Risk of reimprisonment and  rearrest following 1 year or 5 years after release | 11749 offenders convicted of an  indictable offense between October 1976-November 1977 | Holdout validation with training  (n=5875) and testing (n=5874) sets | LR  CT  Iterative LR  ICT | Prison 1 year: 69.3%-83.7%  Prison 5 years: 66.5%-82.5%  Arrest 1 year: 45.6%-68.0%  Arrest 5 years: 54.0%-82.2% | N/A |
| **Silver, 2002** | Official clinical and administrative information of offenders convicted of an indictable offense | Recidivism (imprisonment within 1 and 5 years, and arrest within 1 and 5 years | 11749 offenders convicted of an indictable offense between October 1976-November 1977 | Divided data into 10 subsamples, where 1 was used to construct the risk assessment model and 9 were used for cross-validation | LR ICT  Feature selection using forward stepwise logistic regression | N/A | Model 1-10  Prison 1 year: 0.77-0.85 AUC  Prison 5 years: 0.73-0.78 AUC  Arrest 1 year: 0.73-0.76 AUC  Arrest 5 years: 0.73-0.77 AUC  Multiple Models - full  Prison 1 year: 0.89 AUC  Prison 5 years: 0.81 AUC  Arrest 1 year: 0.79 AUC  Arrest 5 years: 0.79 AUC  Multiple Models - reduced  Prison 1 year: 0.90 AUC  Prison 5 years: 0.81 AUC  Arrest 1 year: 0.78 AUC  Arrest 5 years: 0.80 AUC |
| **Stalans, 2004** | Clinical and demographic variables obtained through clinical charts and legal records. | Violent recidivism while on probation | 1344 violent offenders on probation | LOOCV; no external validation used | CTA - comparing against a logistic model with and without interaction | CTA 78.6% accuracy  Logistic without interaction  81.8% accuracy  Logistic with interaction  81.8% accuracy | CTA: sensitivity; 88.4% specificity  Logistic without interaction: 9.8% sensitivity; 98.7% specificity  Logistic with interaction :8.84% sensitivity; 98.9% specificity |
| **Vilares, 2017** | fMRI collected during a decision-making task | Mental states (knowledge and recklessness) when committing a hypothetical crime | 40 healthy controls | Double-cross validation; no external validation used | Elastic-Net Regression | AUC of 0.792  average correct classification rate (CCR) of 71% | N/A |
| **Haarsma, 2020** | Tablet-based neuropsychological tests | Criminal recidivism | 730 probationers | Training (80%) and testing (20%) samples | GLM  LDA  k-NN  SVM  GBM  RF  EN | N/A | Testing / validation (RFE model)  GLM: 0.68 AUC  LDA: 0.69 AUC  k-NN: 0.60 AUC  SVM (polynomial): 0.67 AUC  GBM: 0.67 AUC  RF: 0.66 AUC  EN: 0.70 AUC |
| **Delfin 2019** | resting-state regional cerebral blood flow (rCBF) and clinical risk factors | Criminal recidivism | 44 forensic psychiatry patients | Out-of-bag (OOB) error | RF | Accuracy: 82%  Sensitivity: 75%  Specificity: 86% | AUC: 0.81  PPV = 0.73  NPV = 0.86  Note: the dataset was not split into training and testing sets, and OOB error was used as a resampling procedure |
|  |  |  |  |  |  |  |  |
| **OTHER OUTCOMES** | | | | | | | |
| **Monaro, 2018** | Behavioral Measures (mouse-movements during a computerized task) | Malingering of clinical depression | 100 individuals both with and without clinical depression | Holdout validation with training (n=60 and test(n=27) sets | NB  SMO  LMT  RF  Feature selection: Correction based feature selection  Validation: 10-fold cross validation | Accuracy in 10-fold-cross validation (n=60)  Naive Bayes - 80  SMO - 82.5  LMT - 80  Random Forest - 87.5  Accuracy in test set (n=28)  Naive Bayes - 94.4  SMO - 88.9  LMT - 88.9  Random Forest - 94.4 | Note: authors did not report sensitivity, specificity, PPV or NPV |
| **Ponseti, 2012** | fMRI blood oxygen level-dependent signals to child and adult sexual stimuli for each participant | Identification of pedophilia | 24 participants with pedophilia  32 healthy controls | LOOCV | LDA  k-NN | k-NN: 75-91%  LDA: 89-95% | Sensitivity / Specificity  k-NN: 63-88% / 84-94%  LDA: 88-92% / 88-100% |
| **Ponseti, 2015** | Haemodynamic fMRI response to face images of women, girls, men, and boys | Classification of Pedophilia | 24 males diagnosed with pedophilia according to the DSM-IV-R  (11 heterosexual pedophiles, 13 homosexual pedophiles). | LOOCV; no external validation | Fisher's linear discriminant analysis | Mean classification accuracy of 93% | 91% specificity  95% sensitivity |
| **Rosenfeld, 2005** | Official clinical and sociodemographic records from criminal defendants | Stalking behavior | 204 individuals evaluated for crimes related to stalking or obsessional harassment | Jack-knife classification approach of training sample | CART models comprising: Tree regression, Logistic regression, | N/A | Tree regression - AUC .649  Logistic regression - AUC .706 |
| **Mazza, 2018** | Computerized neuropsychological test | Malingering | 175 individuals | 10-fold CV  Hold-out validation with training (70.6%) and testing (29.4%) sets | LR  SVM  NB  RF  LMT | Time-pressure models: 95% accuracy across all models  Non time-pressure models: Accuracy ranged from 75-95% | AUC not reported  Note: it is important to mention that a small testing set was used (n=20), which may yield inflated accuracy. |
| **Pace, 2019** | Test taking effort assessment (b Test) | Malingering | 63 individuals | LOOCV | NB  LR  SL SVM RF | NB: 90.47%  LR: 90.47%  SL: 92.9%  SVM: 88.09%  RF: 90.47% | NB: 0.89 AUC  LR: 0.85 AUC  SL: 0.91 AUC  SVM: 0.88 AUC  RF: 0.89 AUC  Note: sensitivity and specificity not reported. The model also did not separate the data into training and testing sets, as such, model accuracy may be inflated. |
|  |  |  |  |  |  |  |  |

**Abbreviations:**

ANN, Artificial neural networks; APS, adaptive polynomial splines; AUC, Area under the curve; BART, bayesian additive regression trees; BRL, Bivariate logistic regression; CART, Classification and regression trees; CNN, Convolutional neural networks; CTA, Classification Tree Analysis; DNN, Deep neural networks; DSM IV-R, Diagnostics and Statistical Manual, Version IV, Revised; DT, Decision tree; EN, elastic net; FKBP5, FK506 binding protein 51 - a glucocorticoid-related chaperone and immunophilin protein that plays a role in immune system function; implicated in emotional dysregulation; GLM, Generalized Linear Modeling; GBM, Gradient Boosting Machine; HCR-20, Historical, clinical, risk management-20; ICT, Iterative classification tree; k-NN, k-nearest neighbors; LDA, Linear discriminant analysis; LMT, Logistic Model Trees; LOOCV, Leave-one-out cross validation; LR, Logistic regression; MLPNN, Multi-layer perceptron neural network; MNN, Multi-layer neural networks; MVL, Multivariate logistic regression; NB, Naive Bayes; NBR, Negative binomial regression; NGRI, not guilty by reason of insanity; NN, Neural network; NPV, Negative Predictive Value; PPV, Positive Predictive Value; R^2^, Coefficient of determination; RNN, Recurrent neural network; RT, Regression tree; SL, Simple Logistics; STARRS, Army Study to Assess Risk and Resilience in Service members; SMO, Sequential Minimal Optimization; SNP, Single Nucleotide Polymorphism; SR, spline regressions; SVM, support vector machine.

^1^The sample size showed in the table includes only the number of subjects used for the machine learning model development, with subjects used for other purposes, such as statistical analysis, not being included in this number
