# Supplementary material for: Predicting criminal and violent outcomes in psychiatry: a meta-analysis of diagnostic accuracy
Source: Transl Psychiatry. 2022 Nov 9;12:470. doi: 10.1038/s41398-022-02214-3 (PMC9643469; doi:10.1038/s41398-022-02214-3)
Supplement: Supplementary file 3 — Supplementary Table S3 [file 41398_2022_2214_MOESM3_ESM.docx]

| **Authors** | **Classification Task** | **Method to address class imbalance** | **True and False Positive/Negative** | **Performance Metrics** | **95% Confidence Intervals of Accuracy** |
| --- | --- | --- | --- | --- | --- |
| Delfin 2019 | Recidivists (N=16)  Non-recidivists (N=28) | Downsampling of majority class | TP = 12  FP = 4  FN = 4  TN = 24 | Balanced Accuracy = 80.5%  Sensitivity = 75%  Specificity = 86%  False Positive = 14%  False Negative = 25%  Standard Error = 6.3775 | Accuracy: 80.5%  (95% CI: 68.92-94.02%) |
| Kirchhebner, 2020 | Recidivists (N=209)  Non-recidivists (N=135) | None | TP = 193  FP = 29  FN = 39  TN = 83 | Balanced Accuracy = 79.4%  Sensitivity = 83%  Specificity = 74% False Positive = 26%  False Negative = 17%  Standard Error = 2.2168 | Accuracy = 79.4%  (95% CI: 76.04-82.91%) |
| Kirchhebner, 2022 | Violent offenders (N=294)  Non-violent offenders (N=75) | SMOTE | TP = 254  FP = 15  FN = 53  TN = 62 | Balanced Accuracy = 75.84%  Sensitivity = 80.49%  Specificity = 71.19%  False Positive = 28.81%  False Negative = 19.51%  Standard Error = 2.1989 | Accuracy = 75.84%  (95% CI: 71.65-76.43%) |
| Linaker, 1995 | Violent patients (N=32)  Non-violent patients (N=60) | None | TP = 32  FP = 11  FN = 0  TN = 49 | Balanced Accuracy = 90.65%  Sensitivity = 100%  Specificity = 81.3%  False Positive = 18.7%  False Negative = 0%  Standard Error = 3.6403 | Accuracy = 90.65%  (95% CI: 83.79-98.07%) |
| Pflueger, 2015 | Recidivists (N=128)  Non-recidivists (N=131) | None | TP = 108  FP = 18  FN = 20  TN = 113 | Balanced Accuracy = 85%  Sensitivity = 84%  Specificity = 86%  False Positive = 14%  False Negative = 16%  Standard Error = 2.2908 | Accuracy = 85.00%  (95% CI: 80.64-89.61%) |
| Sonnweber, 2021 | Violent offenders (N=294)  Non-violent offenders (N=75) | Oversampling minority class | TP = 214  FP = 28  FN = 80  TN = 47 | Balanced Accuracy = 67.82%  Sensitivity = 72.73%  Specificity = 62.92%  False Positive = 37.08%  False Negative = 27.27%  Standard Error = 3.4872 | Accuracy = 67.82%  (95% CI: 61.32-75.01%) |
| Thomas, 2005 | Violent at follow-up (N=158)  Non-violent at follow-up (N=622) | None | TP = 30  FP = 25  FN = 128  TN = 597 | Balanced Accuracy = 57.5%  Sensitivity = 19%  Specificity = 96%  False Positive = 4%  False Negative = 81%  Standard Error = 1.8035 | Accuracy = 57.50%  (95% CI: 51.20-64.57%) |
| Wang, 2020 | Violent at follow-up (N=103)  Non-violent at follow-up (N=172) | None | TP = 65  FP = 117  FN = 38  TN = 55 | Balanced Accuracy = 47%  Sensitivity = 63%  Specificity = 32%  False Positive = 68%  False Negative = 37%  Standard Error = 0.4 | Accuracy = 47%  (95% CI: 46.54-47.47%) |
| Watts, 2021 | Violent & Non-violent offenses (N=1116)  Sexual offenses (N=124)  *Following downsampling*:  Violent & Non-violent offenses (N=248)  Sexual offences (N=74) | Downsampling of the majority class | *Metrics following downsampling*:  TP = 61  FP = 99  FN = 12  TN = 149 | Balanced Accuracy = 71.58%  Sensitivity = 83.15%  Specificity = 60.00%  False Positive = 40%  False Negative = 16.85%  Standard Error = 2.3928 | Accuracy = 71.58%  (95% CI: 67.04-76.43%) |
|  | | | | |  |

**Supplementary Table S3: Confusion Matrices of Classification Models**

False positive rate is calculated as 1-specificity, while false negative is calculated as 1-sensitivity. Standard error was calculated by subtracting the upper bound of the 95% CI from the lower bound, and dividing by 3.92 (upper bound - lower bound)/3.92. This standard error calculation was used for all studies, apart from Wang et al. 2020, which reported standard error as 0.4. 95% confidence intervals are reported as calculated using an inverse variance method within a random effects model. Additionally, confusion matrices were provided according to the method used to address class imbalance, where applicable. It is important to note that none of the included studies reported the true positives/true negatives and false positives/false negative rates, and the numbers indicated in the table reflect calculations based on the prevalence, sensitivity, specificity, and total sample size.
