# Supplementary figures and images for: Predicting criminal and violent outcomes in psychiatry: a meta-analysis of diagnostic accuracy
Source: Transl Psychiatry. 2022 Nov 9;12:470. doi: 10.1038/s41398-022-02214-3 (PMC9643469; doi:10.1038/s41398-022-02214-3)

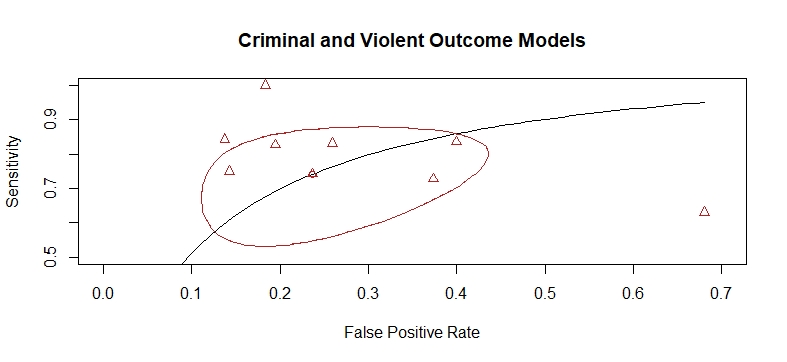


**Supplementary Figure S1: False Positive Rate Against Sensitivity Across Studies**

Supplement: Supplementary file 4 — Supplementary Figure S1 [file 41398_2022_2214_MOESM4_ESM.docx]
