# Supplementary material for: Predicting criminal and violent outcomes in psychiatry: a meta-analysis of diagnostic accuracy
Source: Transl Psychiatry. 2022 Nov 9;12:470. doi: 10.1038/s41398-022-02214-3 (PMC9643469; doi:10.1038/s41398-022-02214-3)
Supplement: Supplementary file 5 — Supplementary Material [file 41398_2022_2214_MOESM5_ESM.docx]

1. **Search Filter**

***Scopus***

((artificial AND intelligence) OR (supervised AND machine AND learning) OR (k-nearest AND neighbors) OR (decision AND trees) OR (naive AND bayes) OR (random AND forest) OR (gradient AND boosting) OR (elastic AND net) OR (support AND vector AND machine) OR (relevance AND vector AND machine) OR (Latent Class Analysis) OR (Neural Networks)) AND ((commitment AND of AND mentally AND ill) OR (insanity AND defense)) OR ((criminals) OR (schizophrenia) OR (schizophrenia AND spectrum AND other AND psychotic AND disorders) OR (psychotic AND disorders) OR (forensic AND psychiatry))

Results: 6531

Search Date: 2022-04-18

***PubMed***

(((((((((("Artificial Intelligence/classification"[Mesh] OR "Artificial Intelligence/methods"[Mesh])) AND ("Supervised Machine Learning/classification"[Mesh]) OR "Commitment of Mentally Ill/statistics and numerical data"[Mesh])) OR ( "Insanity Defense/classification"[Mesh] OR "Insanity Defense/statistics and numerical data"[Mesh])) AND ("Criminals/classification"[Mesh] OR "Criminals/statistics and numerical data"[Mesh])) AND ("Schizophrenia/classification"[Mesh] OR "Schizophrenia/diagnostic imaging"[Mesh] OR "Schizophrenia/statistics and numerical data"[Mesh])) AND ("Schizophrenia Spectrum and Other Psychotic Disorders/classification"[Mesh] OR "Schizophrenia Spectrum and Other Psychotic Disorders/diagnosis"[Mesh] OR "Schizophrenia Spectrum and Other Psychotic Disorders/statistics and numerical data"[Mesh] )) AND ( "Psychotic Disorders/diagnosis"[Mesh] OR "Psychotic Disorders/statistics and numerical data"[Mesh] )) OR ("Forensic Psychiatry/classification"[Mesh] OR "Forensic Psychiatry/statistics and numerical data"[Mesh]))

Results: 1613

Search Date: 2022-04-18

***Web of Science***

(TS=(Artificial Intelligence) OR TS = (Supervised Machine Learning) OR TS = (Deep Learning) OR AB = (Support Vector Machin*) OR AB = (Relevance Vector Machin*) OR AB = (Random Forest) OR AB = (Decision Tree*) OR AB = (Gradient Boost*) OR AB = (Extreme Boost*) OR AB = (Elastic Net) OR AB = (Logistic Regression) OR AB = (Naive Bayes) OR AB= (Neural Network*) OR TS = (Expert System*) OR TS = (Latent Class Analys*)) AND (TS = (Forensic Psych*) OR TS = (Commitment of Mentally Ill) OR TS = (Insanity Defence) OR TS = (Crimin*) OR TS = (Offend*))

Results: 4792

Search Date: 2022-04-18

Total records(before duplicate removal): 12936

Total records(duplicates removed): 12420

**2. Quality assessment instrument development**

We formed a group of multidisciplinary researchers from the fields of Neuroscience, Psychiatry, and Computer Science to develop a time efficient and practical assessment strategy to evaluate the quality of machine learning based healthcare research. For that purpose, we attempted to capture the reliability of the results presented in a given study and identify practical ways that methodology may be improved.

This comprised nine methodological features, including sample representativeness, confounding variables, and outcome assessments, which were judged to be the most clinically pertinent components in machine learning-based healthcare research. Relevant considerations of each methodological feature are discussed in further detail in the next sections. The six remaining dimensions assess the quality and specific components of the machine learning approach that were used in a given study. In summary, this entails the algorithm or framework used, evidence that hyper-parameter optimization and feature selection procedures were used, whether authors provided details on how missing data and class imbalance problems were handled, the accuracy of a given model, and finally whether the model performance was tested in unseen data. These dimensions were qualitatively evaluated according to the information in section 3.

**3. Quality assessment instrument domains**

| Methodological Feature | Considerations |
| --- | --- |
| 1. Representativeness of  the sample | Was the study representative of the heterogeneity observed in the target population? If not, was this related to the sampling method, insufficient sample size or inclusion/exclusion criteria? |
| 2. Confounding variables | Did the study control for the most relevant confounding variables? If so, were covariates assessed using subjective or objective measures? |
| 3. Outcome assessment | How were outcome measures assessed: A. Independent blind assessment (✓)  B. Secure record (e.g. surgical records) (✓)  C. Interview not blinded, self-report or medical record  D. No description |
| 4. Algorithm selection | Was the machine learning algorithm used to analyse the data clearly described and appropriate? |
| 5. Feature selection | Did the study describe both feature selection and hyperparameter tuning? Which metrics were used? |
| 6. Class imbalance | Did the authors address the class imbalance problem? Which method was used? |
| 7. Missing data | Did the study describe how the authors handled missing data, including whether they were inputted or removed? |
| 8. Performance/accuracy | Were the following performance metrics included for classification studies?   1. Accuracy 2. Sensitivity 3. Specificity 4. AUC 5. PPV/NPV 6. 95% Confidence intervals of performance metrics   Or, alternatively, were one of the following performance metrics included for regression studies?   1. Mean-squared error 2. Mean-absolute error 3. Root-mean-squared error |
| 9. Testing/validation | Was the test dataset "unseen" in regard to model training? Was the model tested on a hold-out or an external dataset? |

*3.1. Representativeness of the sample*

Machine learning models can deal with large amounts of data and the problem of heterogeneity. Therefore, there is less of a need to be restrictive with inclusion and exclusion criteria. Here, we evaluated whether the sample selected by the authors reflected the real population being studied. When the sample did not reflect the population being studied, we evaluated if it was because (1) the sampling methods were not appropriate, (2) the sample was not large enough to represent the population or (3) the inclusion and exclusion criteria restricted the individuals in the study.

*3.2. Confounding Variables*

To adequately control for confounding variables in machine learning, we need to ensure that they will have a similar effect across the entirety of the sample. To achieve this, randomization is used throughout the analysis. More specifically, training and testing datasets are randomised using resampling techniques, and the analysis is often repeated with different parameters and learning decisions (parameter tuning). Using the aforementioned criteria, we evaluated whether the authors controlled for confounding variables.

*3.3. Outcome assessment*

How an outcome is defined has several important implications in a predictive model. Depending on the question or problem, a classification task may be appropriate, which uses a categorical outcome, or a regression task may be more relevant, which has a continuous numeric outcome. A clinical instrument or questionnaire, for example, can be used as a numeric score or it can be transformed into a categorical outcome by using a cut-off. We evaluated how authors assessed these outcomes, considering (1) independent blind assessments and secure records as high quality, (2) unblinded interview, self-report or medical record as lower quality and (3) when no description was available.

*3.4. Algorithm selection*

There are several algorithms to choose from, with each relying on slightly different assumptions of the underlying data. Broadly speaking, there are linear (logistic regression, linear support vector machine), non-linear (Naive Bayes, K-Nearest Neighbours, Learning Vector Quantization), tree-based (decision trees, random forest, xgboost) and neural network (convolutional neural network, multilayer perceptrons) models, although others exist. Certain algorithms may be better suited to particular problems. For example, tree-based models such as random forest may be better suited to datasets with multicollinearity among features than linear-based models such as logistic regression. However, regularisation parameters can be used in linear-based models (such as L2 regularisation) to account for issues such as this. Nevertheless, it is often difficult to determine beforehand which algorithms will lead to the highest model performance. Therefore, it is often a good strategy to compare the model performance of several algorithms. In this item, we evaluated whether the authors used an algorithm that is commonly used for the specific type of dataset, if several algorithms were compared, and if hyperparameter tuning was used.

The appropriateness of a machine learning algorithm was determined based on whether the specific data used in model development was congruent or incongruent with the strengths and limitations of the specific algorithm. For example, if a Gaussian process model was used, which is a non-sparse algorithm that loses efficiency in high dimensional spaces, in conjunction with a high-dimensional dataset, this algorithm would be deemed inappropriate for the input data. Conversely, Naive Bayes, which works well with high dimensional data would be considered an appropriate algorithm in such cases. Another example of an inappropriate model would be the use of convolutional neural networks for structural and tabular style datasets, as such algorithms are better suited to unstructured datasets. In cases where authors included both appropriate and inappropriate algorithms during model development, this consideration is scored with a “B”, alongside an asterisk to indicate which algorithms were inappropriate and why. Studies which only utilised one algorithm during model development that was deemed inappropriate received a score of “C”. Furthermore, studies are scored with a “B” if they did not compare multiple algorithms during model development, and were scored as an “A” if they compared multiple algorithms that were deemed appropriate based on the candidate feature set.

*3.5. Feature selection*

A common problem in machine learning studies is the so-called small-n-large-p problem, also known as the curse of dimensionality, which occurs when there are more variables than examples in a dataset. Machine learning models created using these datasets are more prone to overfitting, which often results in overinflated performance in a training dataset, but much poorer performance in an external testing dataset. In addition, some algorithms cannot deal with more dimensions than examples. Highly correlated variables can also introduce more importance to a specific characteristic, decreasing the importance of the remaining variables. To circumvent these issues, a proper feature selection procedure, when applicable, should be done prior to training or as part of the training procedure, such as it happens in embedded methods. The feature selection can be knowledge-driven or data-driven. In this item, we examined if the study used a proper feature selection (if applicable).

*3.6. Class imbalance*

Class imbalance occurs when the distribution of the outcome classes is highly unbalanced, i.e., when one outcome occurs much more frequently than the other one. This may result in a model with high accuracy but with very little clinical utility. For example, let us suppose that we have 99 occurrences of non-violence in our dataset and only 1 occurrence of a violent incident. Even if our model has 99% accuracy, it is useless if the model cannot detect the one violent incident with high accuracy. In this item, we evaluated whether there was a class imbalance in the sample and if this problem was correctly addressed. This can be done using a series of methods, including (1) changing the metric of performance (accuracy, for example, is a poor form of evaluating imbalanced data sets; (2) resampling the data set by artificially increasing it (oversampling) or by removing examples from the majority class to create a more balanced data set (undersampling); (3) by generating more data with algorithms such as the Synthetic Minority Over-Sampling Technique (SMOTE); (4) by choosing algorithms that deal better with unbalanced classes, such as CART or random forests; (5) by using penalised models; or (6) by using anomaly and change detection.

*3.7. Missing data*

It is critical to handle missing data since a number of algorithms cannot process incomplete data sets. Furthermore, it is also necessary to use an adequate imputation method to avoid introducing bias, which would otherwise lead to false conclusions if not addressed. It is important to report the amount of missing data in each variable, if these cases were excluded, or if the authors used an algorithm to input data and which algorithm/technique was used. Ideally, authors should provide a visual distribution of the patterns of missing data, such as aggregation plots, spinogram/spineplots, mosaic plots, etc. All these factors were evaluated in this section.

*3.8. Performance/accuracy*

Here, we evaluate whether the authors reported all relevant results and if they used the appropriate metrics. Studies informing only partial metrics may mask bias and flaws of the method, preventing the reader from fully understanding the relevance of the model.

*3.9. Testing/Validation*

We can divide the machine learning process into three main components: training, validation, and testing. A training set allows the algorithm to learn and develop a predictive model. The validation set contains unseen data and is used to control for overfitting. Frequently, the same dataset is divided into training and validation sets. After a model is trained and validated, and shows consistent performance in both these steps, the model can be applied in an external and independent testing set. This allows us to see if the model can be generalised outside of the original sample. Some validation methods include holdout validation, k-fold, and leave one out cross validation.

A model that shows good performance in the training set but performs significantly poorer in the validation step is most likely due to overfitting - which occurs when the model relies more on the specific nuances and noise of the training dataset, resulting in poor accuracy in unseen data. In this item, we evaluated whether the authors properly tested and validated their models by taking steps to improve its generalizability. It is important to highlight that the use of cross-validation to evaluate performance should be discouraged when the data is large enough for a training-test split. Furthermore, the size of the test set should be sufficiently large for accuracy and other metrics to be estimated with high reliability.

**4. R Scripts**

**4.1 mean_accuracy_meta-analysis.R**

library(dmetar)

library(esc)

library(tidyverse)

library(dplyr)

library(meta)

criminal_df = data.frame(observations = c(44, 344, 369, 92, 259, 369, 219, 780, 322),

study = c("Defin 2019", "Kirchhebner, 2020", "Kirchhebner, 2022", "Linaker, 1995", "Pflueger, 2015", "Sonnweber, 2021", "Thomas, 2005", "Wang, 2020", "Watts, 2021"),

mean_accuracy = c(80.5, 79.4, 75.84, 90.65, 85.00, 67.82, 57.50, 47.00, 71.58),

standard_error = c(6.3775, 2.2168, 2.1989, 3.6403, 2.2908, 3.4872, 1.8035, 0.40, 2.3928),

standard_deviation = c(42.30, 32.46, 42.23, 34.91, 36.86, 66.98, 50.36, 6.63, 42.93))

criminal_mean = metamean(n = criminal_df$observations,

mean = criminal_df$mean_accuracy,

sd = criminal_df$standard_deviation,

studlab = criminal_df$study,

sm = "MLN",

level = 0.95,

pval = TRUE,

Q = TRUE,

pval.random = TRUE,

fixed = FALSE,

random = TRUE,

method.tau = "REML",

hakn = TRUE,

se.tau = TRUE,

title = "Criminal and Violent Outcomes in Psychiatry")

# Removing Linaker 1995 %

subset_criminal = subset(criminal_df, study != "Linaker, 1995")

subset_mean = metamean(n = criminal_df$observations,

mean = criminal_df$mean_accuracy,

sd = criminal_df$standard_error,

studlab = criminal_df$study,

sm = "MLN",

pval = TRUE,

fixed = FALSE,

random = TRUE,

method.tau = "REML",

hakn = TRUE,

title = "Criminal and Violent Outcomes in Psychiatry")

forest.meta(criminal_mean,

sortvar = TE,

print.tau2 = TRUE,

print.I2 = FALSE,

print.Q = FALSE,

leftlabs = c("Author", "g", "SE"),

level.com = 0.95,

digits = 2)

**4.2. crime_mada.R**

## Import packages ##

library("meta", "mada")

## creating forest plots using mada ##

crime_madad = mada::madad(TP = c(12, 193, 254, 32, 108, 214, 30, 65, 61),

FP = c(4, 29, 15, 11, 18, 28, 25, 117, 99),

FN = c(4, 39, 53, 0, 20, 80, 128, 38, 12),

TN = c(24, 83, 62, 49, 113, 47, 597, 55, 149),

level = 0.95,

correction = 0.5,

correction.control = "all",

method = "wilson",

yates = TRUE)

crime_madad$names = c("Delfin, 2019",

"Kirchhebner, 2020",

"Kirchhebner, 2022",

"Linaker, 1995",

"Pflueger, 2015",

"Sonnweber, 2021",

"Thomas, 2005",

"Wang, 2020",

"Watts, 2021")

# Sensitivity and specificity #

crime_madad$sens

crime_madad$spec

plot.new()

par(fig = c(0, 0.5, 0, 1), new = TRUE)

mada::forest(crime_madad, type = "sens", xlab = "Sensitivity", snames = crime_madad$names, main = "Model Sensitivity" )

par(fig = c(0.5, 1, 0, 1), new = TRUE)

mada::forest(crime_madad, type = "spec", xlab = "Specificity", snames = crime_madad$names, main = "Model Specificity" )

# Negative and positive likelihood ratios

plot.new()

par(fig = c(0, 0.5, 0, 1), new = TRUE)

mada::forest(crime_madad, type = "negLR", xlab = "negative likelihood ratio", snames = crime_madad$names, main = "Likelihood Ratios" )

par(fig = c(0.5, 1, 0, 1), new = TRUE)

mada::forest(crime_madad, type = "posLR", xlab = "positive likelihood ratio", snames = crime_madad$names, main = "Likelihood Ratios" )

# Diagnostic Odds Ratio #

par(mfrow=c(1,1))

mada::forest(crime_madad, type = "DOR", xlab = "log diagnostic odds ratio", snames = crime_madad$names, main = "Criminal and Violent Models" )

# Diagnostic Meta-Analysis with the proportional hazards model approach of Holling et al. (2012) #

# First need to create a dataframe #

crime_df = data.frame(TP = c(12, 193, 254, 32, 108, 214, 30, 65, 61),

FP = c(4, 29, 15, 11, 18, 28, 25, 117, 99),

FN = c(4, 39, 53, 0, 20, 80, 128, 38, 12),

TN = c(24, 83, 62, 49, 113, 47, 597, 55, 149))

crime_phm = mada::phm(crime_df,

TP = "TP",

FN = "FN",

FP = "FP",

TN = "TN",

correction = 0.5,

correction.control = "all",

hetero = TRUE,

estimator = "AMPLE")

# Fit the bivariate model of Reitsma et al. (2005) and extensions #

crime_reitsma = mada::reitsma(crime_df,

TP = "TP",

FN = "FN",

FP = "FP",

TN = "TN",

formula = NULL,

alphasens = 1,

alphafpr = TRUE,

correction = 0.5,

correction.control = "all",

method = "reml"

)

cr.crime = mada::ROCellipse(crime_reitsma, correction = 0.5, level = 0.95, method = "wilson", extrapolate = TRUE, predict = TRUE)

cr.sroc = mada::sroc(crime_reitsma, fpr = 1:99/100, type = "ruttergatsonis", return_function = FALSE)

plot(crime_reitsma, xlim = c(0, 0.7), ylim = c(.5, 1),

main = "Criminal and Violent Outcome Models", col = "#B22222")

mada::ROCellipse(crime_reitsma, lty = 2, pch = 2, add = TRUE, col = "#B22222")

points(mada::fpr(crime_df), mada::sens(crime_df), pch = 2, cex = 1, col = "#B22222")

# Meta-Analysis of univariate measures of diagnostic accuracy #

crime_madauni_DOR = mada::madauni(crime_df,

type = "DOR",

method = "DSL",

suppress = TRUE)

crime_posLR = mada::madauni(crime_df,

type = "posLR",

method = "DSL",

suppress = TRUE)

crime_negLR = mada::madauni(crime_df,

type = "negLR",

method = "DSL",

suppress = TRUE)

# Forest plot #

mada::forest(crime_madauni_DOR,

log = TRUE )

# Calculating AUC and 95% CI #

summary_AUC = AUC_boot(TP = c(12, 193, 254, 32, 108, 214, 30, 65, 61),

FP = c(4, 29, 15, 11, 18, 28, 25, 117, 99),

FN = c(4, 39, 53, 0, 20, 80, 128, 38, 12),

TN = c(24, 83, 62, 49, 113, 47, 597, 55, 149),

B = 2000,

alpha = 0.95)

# Calculating partial AUC #

crime_AUC = mada::AUC(crime_reitsma, fpr = 1:99/100, level = 0.95, sroc.type = "ruttergatsonis")
